# Down-regulation of KLRB1 is associated with increased cell growth, metastasis, poor prognosis, as well as a dysfunctional immune microenvironment in LUAD

**DOI:** 10.1038/s41598-024-60414-x

**Published:** 2024-05-23

**Authors:** Jiu-Ling Chen, Chuang-Yan Wu, Xiang-Yu Luo, Xue-Ying Wang, Fang-Ming Wang, Xin Huang, Wei Yuan, Qiang Guo

**Affiliations:** 1 Department of Thoracic Surgery, Union Hospital, Tongji Medical College, Huazhong University of Science and Technology, Wuhan, China; 2 Department of Cardiothoracic Surgery, Taihe Hospital, Hubei University of Medicine, Shiyan, China; 3 Department of Basic Medicine, Hubei University of Medicine, Shiyan, China; 4 Department of Thoracic Surgery, People’s Hospital of Dongxihu District, Wuhan, China

**Keywords:** KLRB1, Lung adenocarcinoma, Prognosis, ROC, Disease-specific survival, Computational biology and bioinformatics, Biomarkers

## Abstract

Killer cell lectin-like receptor B1 (KLRB1) is implicated in cancer progression and immunity. In this study, we aimed to evaluate the expression levels of KLRB1 in lung adenocarcinoma (LUAD) and analyze the relationship between KLRB1 expression levels, LUAD progression, and the tumor immune microenvironment. KLRB1 levels in LUAD were analyzed using data from the TCGA and XENA databases. Additionally, the diagnostic values of KLRB1 were analyzed in patients with LUAD. Survival and meta-analyses were employed to investigate the relationship between KLRB1 levels and other prognostic factors in patients with LUAD. Bioinformatics and cellular experiments were used to understand the functions and mechanisms of KLRB1. In addition, correlation analysis was used to investigate the relationship between KLRB1 levels and the immune microenvironment in LUAD. Reduced KLRB1 expression in LUAD was found to positively correlate with tumor size, distant metastasis, pathological stage, age, overall survival, diagnostic value, and disease-specific survival in patients with LUAD (*P* < 0.05). Conversely, increased KLRB1 expression was found to positively correlate with the overall survival and disease-specific survival in patients with LUAD (*P* < 0.05). We also found that the overexpression of KLRB1 can inhibit the proliferation, migration, and invasion of LUAD cells and promote apoptosis. KLRB1 was involved in immune cell differentiation, NF-kB, PD-L1, and PD-1 checkpoint pathways and others. Additionally, KLRB1 expression was linked to tumor purity, stromal, immune, and estimate scores, the levels of immune cells including B cells, CD8^+^ T cells, and CD4^+^ T cells, and immune cell markers in LUAD. Reduced KLRB1 expression has a significant positive correlation with diagnosis, poor prognosis, and immunity to cancer in patients with LUAD. KLRB1 inhibited cell proliferation and migration in patients with LUAD. These results suggest that KLRB1 may serve as a potential therapeutic target in patients with LUAD.

## Introduction

The incidence and mortality of lung cancer remains high^1,2^. Although surgical treatment is expected to yield an excellent prognosis with a considerable 5-year survival rate in patients with early-stage lung cancer, nevertheless, patients with intermediate and advanced stages of lung cancer frequently experience tumor progression and poor survival times. Recent therapeutic advances, notably immunotherapy, have improved the prognosis of patients with lung cancer^3,4^. However, therapeutic breakthroughs for the treatment of moderately advanced lung cancer have been inefficient, and finding therapeutic alternatives to improve the prognoses of patients with advanced lung cancer remains a pressing issue.

Previous studies have established a close relationship between gene expression levels and lung adenocarcinoma (LUAD) progression^5–7^. For instance, serum amyloid A-like 1 (SAAL1), which is overexpressed in LUAD tissues, is associated with shorter overall survival (OS), progression-free interval (PFI), and disease-specific survival (DSS), and poor diagnostic value in patients with LUAD. However, the inhibition of SAAL1 expression delays the proliferation of A549 cells by suppressing cyclin D1 and Bcl-2 protein expression^5^. Therefore, SAAL1 is a promising prognostic biomarker for patients with LUAD. Research confirms that Killer cell lectin-like receptor B1 (KLRB1), an immune gene, is a potential biomarker for cancer^8–14^. For example, it is overexpressed in patients with high-grade glioma (GBM) and IDH wild-type GBM and is associated with GBM progression via its unique effects on T-cell dysfunction^8^. The expression level of KLRB1 is reduced in LUAD tissues and is linked to poor prognosis in patients with LUAD^9^. However, the roles of KLRB1 in LUAD remains unclear. Hence, this study aims to investigate the roles and mechanisms of KLRB1 and its relationship with the immune microenvironment in LUAD and to elucidate its potential as a prognostic marker for patients with LUAD.

## Materials and methods

### Data mining from the Cancer Genome Atlas (TCGA) and XENA databases

The gene expression data from 59 normal tissue samples and 535 cancer tissue samples of patients with LUAD were extracted along with their clinical data consisting of the survival time, survival status, progression time, cause of death, age, gender, clinical stage, T stage, and others, from the TCGA database using the Perl language^15^. Additionally, 288 normal lung tissue samples in the GTEx database were obtained from the XENA database using the Perl language^15^.

### Identification of the expression levels of KLRB1 in LUAD

Expression analysis was performed to determine the expression levels of KLRB1 in 59 normal and 535 LUAD tissues, and in 57 normal and 57 cancer tissues obtained from the same patients. In addition, the expression of KLRB1 were explored in LUAD tissues based on the clinical-pathological characteristics of the patients.

### Determination of the diagnostic and prognostic values of KLRB1 in LUAD

A receiver operating characteristic (ROC) analysis was used to assess the diagnostic value of KLRB1 in normal and cancer tissues^16,17^. In this study, ROC analysis was used to evaluate KLRB1 expression in normal lung tissues and LUAD tissues obtained from the TCGA and XENA databases, and the area under the ROC curve was calculated for each dataset. Furthermore, the KLRB1 expression data of cancer tissues were merged with the prognosis data of patients with LUAD using Perl language. Subsequently, the patients were divided into two groups based on the median value of KLRB1 expression, and the statistical relationship between KLRB1 expression changes and the progression and prognosis of LUAD in patients were investigated using data from the TCGA database. This study utilized data sourced from the Kaplan–Meier plotter and lung cancer explorer (LCE) in the TCGA and GEO databases, and the relationship between KLRB1 expression changes and cancer prognosis and progression in patients with LUAD were determined using K–M survival analysis and meta-analysis^18^.

### Genes co-expressed with KLRB1

The correlation coefficient was used to indicate the strength of the correlation between two genes. The Pearson correlation method was employed on LUAD tissue samples to determine the genes co-expressed with KLRB1, and as per literature reports, those with a correlation coefficient greater than 0.6 were considered significantly correlated with KLRB1^19^.

### Functions, mechanisms and networks associated with the genes co-expressed with KLRB1

Gene Ontology (GO) and Kyoto Encyclopedia of Genes and Genomes (KEGG) analyses, available in the Metascape database^20^, are commonly utilized to explore the functions and mechanisms of multiple genes^19^. To investigate the functions and mechanisms of the genes co-expressed with KLRB1, the genes were added to Metascape, and the species was designated as ‘human’. Additionally, the data from the TCGA database in the Cancer Multi-Omics Atlas Integration Project (CAMOIP) was utilized to explore the mechanisms associated with the KLRB1 gene. Gene Set enrichment analysis (GSEA) was performed and the statistical significance threshold was set at a adjusted value of *P* < 0.05. Finally, the protein network of the genes co-expressed with KLRB1 was constructed using the STRING database, and the visualization was enhanced using the Cytoscape software^20^.

### Cell culture and construction of KLRB1-overexpressing LUAD model

RPMI-1640 medium containing 10% fetal bovine serum (FBS) and 1% antibiotics was prepared in advance. A549 and H1299 cells were purchased from the Chinese Academy of Sciences, Shanghai, and cultured in a CO_2_ incubator at 37 °C and CO_2_ concentration of 5%. The culture medium was changed regularly and replaced with a fresh medium, taking into consideration the cell adhesion and growth rate. To achieve KLRB1 overexpression, the KLRB1 gene was introduced into LUAD cells using an adenovirus vector. The KLRB1 overexpression model was constructed as reported previously^14^ and verified through western blotting and real-time quantitative PCR. The primers for the KLRB1 gene were: ATCTCTTCCTCGGGATGTCTGTCAG (Forward primer) and AGGATGTCACTGAAACACTCAACCC (Reverse primer). Western blotting was performed as per the standard methodology using a KLRB1 antibody at a concentration of 1:500.

### Cell counting kit-8

CCK-8 is a widely utilized reagent for assessing cell viability and evaluating the proliferative capacity of LUAD cells. LUAD cells were suspended in an appropriate culture medium and seeded in a 96-well plate at a suitable cell density. A549 and H1299 cells were cultured in an incubator at 37 °C and CO_2_ concentration of 5%. At specified time points (0, 24, 48, and 72 h), 10 μl of CCK-8 reagent was added to each well of the 96-well plate, which was then incubated in a CO_2_ incubator. Following a definite incubation period, the optical density of each well was measured utilizing a microplate reader.

### Cell apoptosis

The LUAD cells from both the control and the KLRB1-overexpression groups were cultured until they reached an optimal cell number and growth condition. Subsequently, the LUAD cells in the culture dishes were harvested, subjected to centrifugation to discard the medium and other impurities, and resuspended in an appropriate buffer. The cells were then stained using Annexin V and propidium iodide (PI) following the manufacturer's instructions, using the recommended concentration and duration of incubation. Cell suspension was obtained either through filtration or centrifugation, and flow cytometry analysis was employed, which enabled the exploration of the excitation, detection, recording, and analysis of fluorescent signals emitted by the dyes.

### Cell invasion

Transwell chambers were soaked in a sterile culture medium to wet the filter membrane. LUAD cells from the control and the KLRB1-overexpression groups were cultured until they reached an appropriate cell number and growth state. The cells in the culture dishes were collected, centrifuged, and resuspended in an appropriate buffer. About 200 μl of the LUAD cell suspension was added to the upper chamber of the Transwell apparatus. The culture dish containing the Transwell chambers was placed in an incubator at 37 °C to provide the appropriate temperature and atmospheric conditions. Analysis and quantification of the number of cells crossing the filter membrane were performed by fixation and staining.

### Cell migration

LUAD cells from both the control and the KLRB1 overexpression groups were cultured until they reached the desired cell number and growth state. To create a consistent wound, a straight-line scratch was made on the cell monolayer in the 6-well plate using a fine pipette tip. The cells in the culture dish were washed several times with sterile PBS or culture medium to eliminate the free cells and cell debris. The 6-well plate was incubated in an incubator at 37 °C to achieve optimal temperature and atmospheric conditions. The progression of wound healing was observed and recorded at specified time intervals. Image analysis software was employed to measure the extent of cell migration and statistical analysis was performed.

### Identification of the relationship between KLRB1 and the immune microenvironment

Cancer tissues obtained from cancer patients were evaluated for their immune, stromal, and estimate scores, and the expression levels of immune cell types, including Tem, CD56bright NK cells, aDC, CD8^+^ T cells, cytotoxic T cells, dendritic cells (DC), eosinophils, B cells, iDC, mast cells, neutrophils, CD56dim NK cells, macrophages, NK cells, pDC, T cells, T helper cells, Tcm, TFH, Tgd, Th1 cells, Th17 cells, Th2 cells, and TReg, using the ESTIMATE and single-sample gene set enrichment analysis (ssGSEA)^21–23^. Pearson correlation analysis was employed to investigate the relationship between KLRB1 and the immune microenvironment, with the statistical significance criterion set at *P* < 0.05. Additionally, the patients were grouped based on the median of the KLRB1 expression levels to determine the statistically significant differences among the immune, stromal, and estimate scores, and the different immune cell expression levels.

### TIMER database

The TIMER database is a web-based cancer database that has been leveraged to analyze the relationship between individual genes and pan-cancer^24,25^. In this study, correlation analyses were conducted to visualize the relationship between KLRB1 levels, tumor purity, and the levels of B cells, CD8^+^ T cells, CD4^+^ T cells, macrophages, neutrophils, and DC, with a significance criterion of *P* < 0.05. Additionally, the correlation analysis module in the TIMER database was used to elucidate the relationship between KLRB1 levels and immune cell markers, with the significance criterion set at *P* < 0.05.

### Identification of the relationship between KLRB1 and immune cell markers

The GEPIA database contains normal and cancer tissue data of patients with cancer sourced from the TCGA and XENA databases^20,24^. In this study, to verify the relationship between KLRB1 levels and immune cell markers, normal and cancer tissue data of patients with LUAD were sourced from the TCGA database.

### Statistical analysis

In this study, the t-test was utilized to explore the expression levels of KLRB1 in LUAD tissues and to investigate the potential relationship between KLRB1 levels at the time of diagnosis and the prognosis of patients with LUAD, which were determined using ROC analysis and survival analysis performed in GraphPad Prism and SPSS software, respectively. Additionally, Pearson correlation was employed to assess the relationship between KLRB1 and the immune microenvironment, with a significance criterion set at *P* < 0.05.

## Results

### KLRB1 expression is significantly down-regulated in LUAD tissues

**Figure 1:**
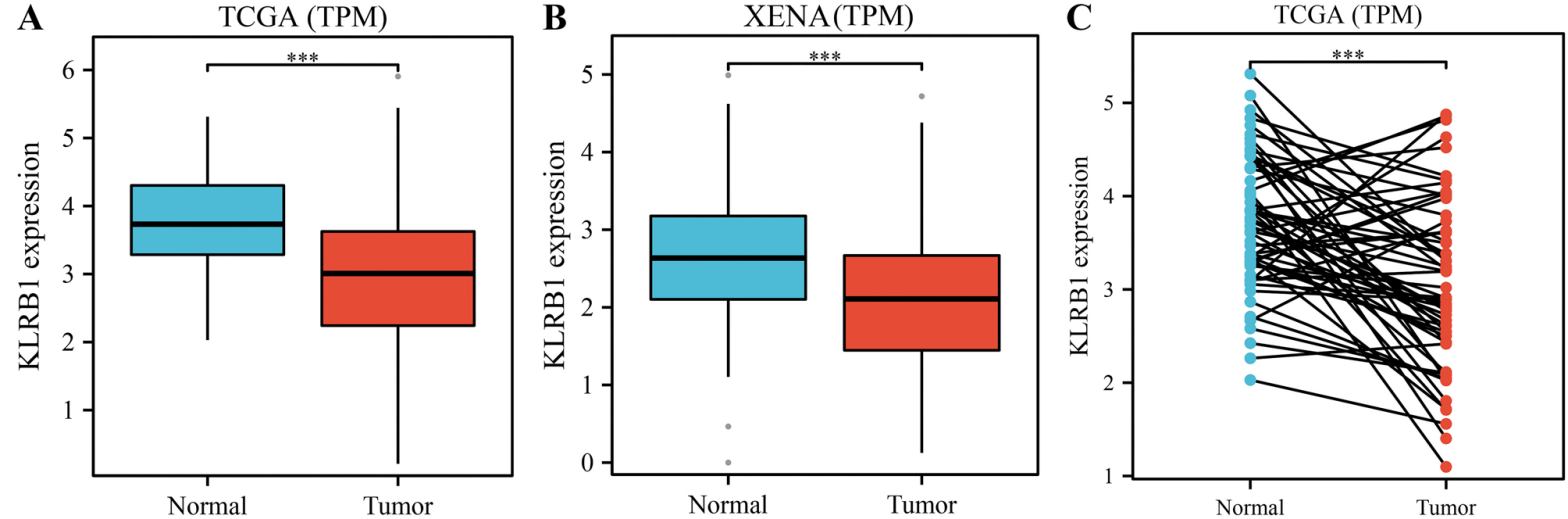
Down-regulation of the expression of KLRB1 in LUAD tissues. (**A**) Data from the TCGA database; (**B**) Data from the TCGA and XENA databases; (**C**) Paired data from the TCGA database. *LUAD* lung adenocarcinoma, *TCGA* The Cancer Genome Atlas, *GTEx* genotype-tissue expression.

**Figure 2:**
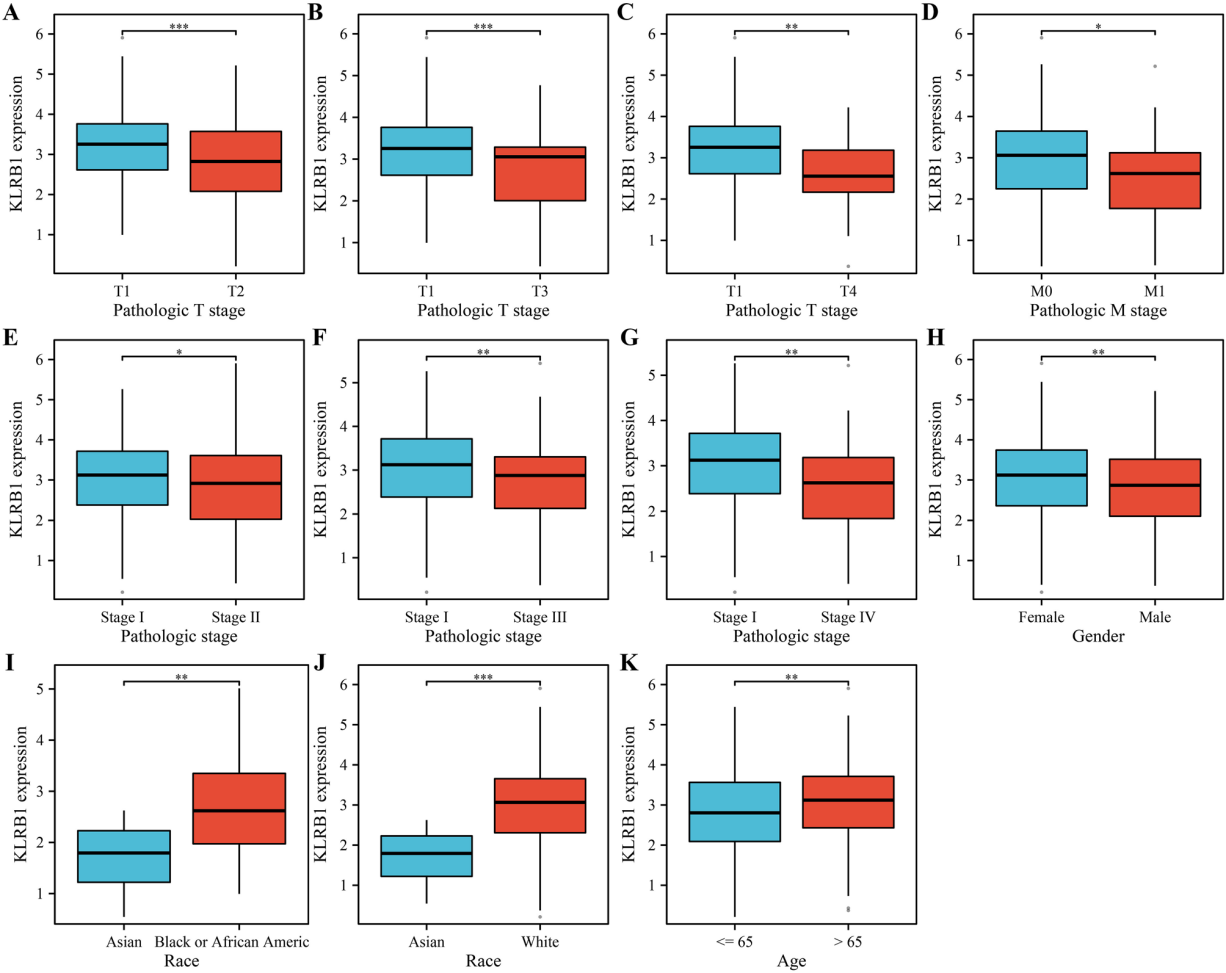
The expression levels of KLRB1 in LUAD tissues. (**A**) T1 versus T2; (**B**) T1 versus T3; (**C**) T1 versus T4; (**D**) M0 versus M1; (**E**) Stage I versus II; (**F**) Stage I versus III; (**G**) Stage I versus IV; (**H**) Female versus Male; (**I**) Asian versus Black or African; (**J**) Asian versus White; (**K**) Age ≤ 65 versus > 65. *LUAD* lung adenocarcinoma, *OS* overall survival, *DSS* disease-specific survival.

Compared with normal tissues from the TCGA and XENA databases, KLRB1 expression levels were significantly down-regulated in unpaired LUAD tissues (Fig. 1A,B) and paired LUAD tissues (Fig. 1C). Additionally, our analysis showed that KLRB1 expression levels were significantly down-regulated in the cancer tissues of patients with T2, T3, and T4 stages of LUAD, compared to those of patients with T1 stage LUAD (Fig. 2A–C). Similarly, KLRB1 expression levels were significantly down-regulated in the cancer tissues of patients with M1 stage LUAD compared to those of patients with M0 stage LUAD (Fig. 2D). Likewise, KLRB1 expression levels were significantly down-regulated in the cancer tissues of patients with stage II, III, or IV LUAD compared to those of patients with stage I LUAD (Fig. 2E–G). Moreover, we found that KLRB1 expression levels were significantly down-regulated in male patients with LUAD compared to those of female patients with the same disease (Fig. 2H). We also observed that KLRB1 expression levels were significantly down-regulated in the cancer tissues of Asians with LUAD compared to those of Caucasian or African-American patients (Figs. 2I,J). Additionally, we found that KLRB1 expression levels were significantly down-regulated in the cancer tissues of patients over the age of 65 compared to those of patients aged 65 or younger (Fig. 2K).

### Reduced expression of KLRB1 is associated with the diagnosis and poor prognosis of patients with LUAD

**Figure 3:**
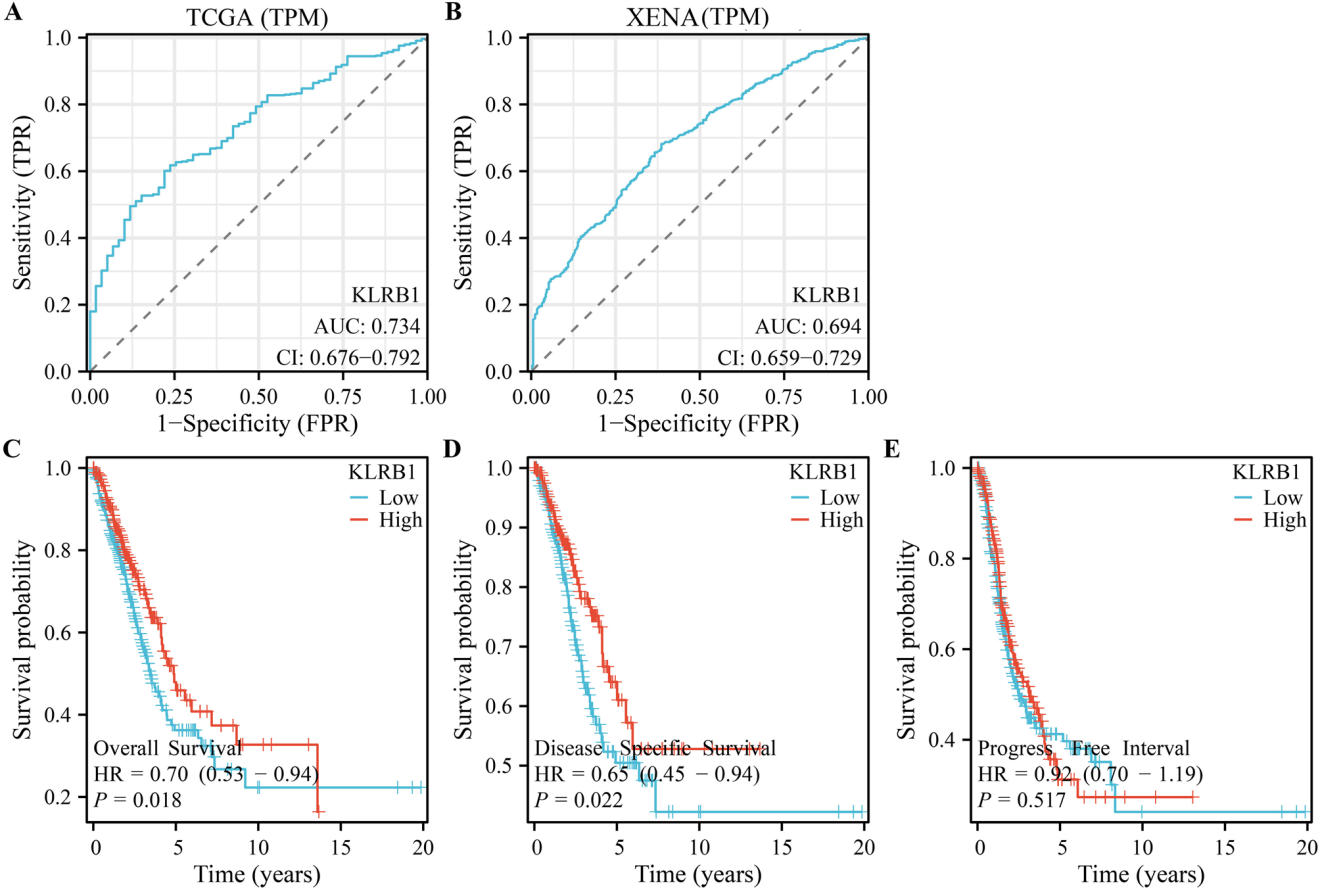
ROC and survival analyses depicting the diagnostic and prognostic effects of KLRB1 in LUAD. (**A**) Data from TCGA database; (**B**) Data from XENA database; (**C**–**E**) Prognostic data from TCGA database. *LUAD* lung adenocarcinoma, *OS* overall survival, *DSS* disease-specific survival, *TCGA* The Cancer Genome Atlas, *PFI* progression-free interval.

**Figure 4:**
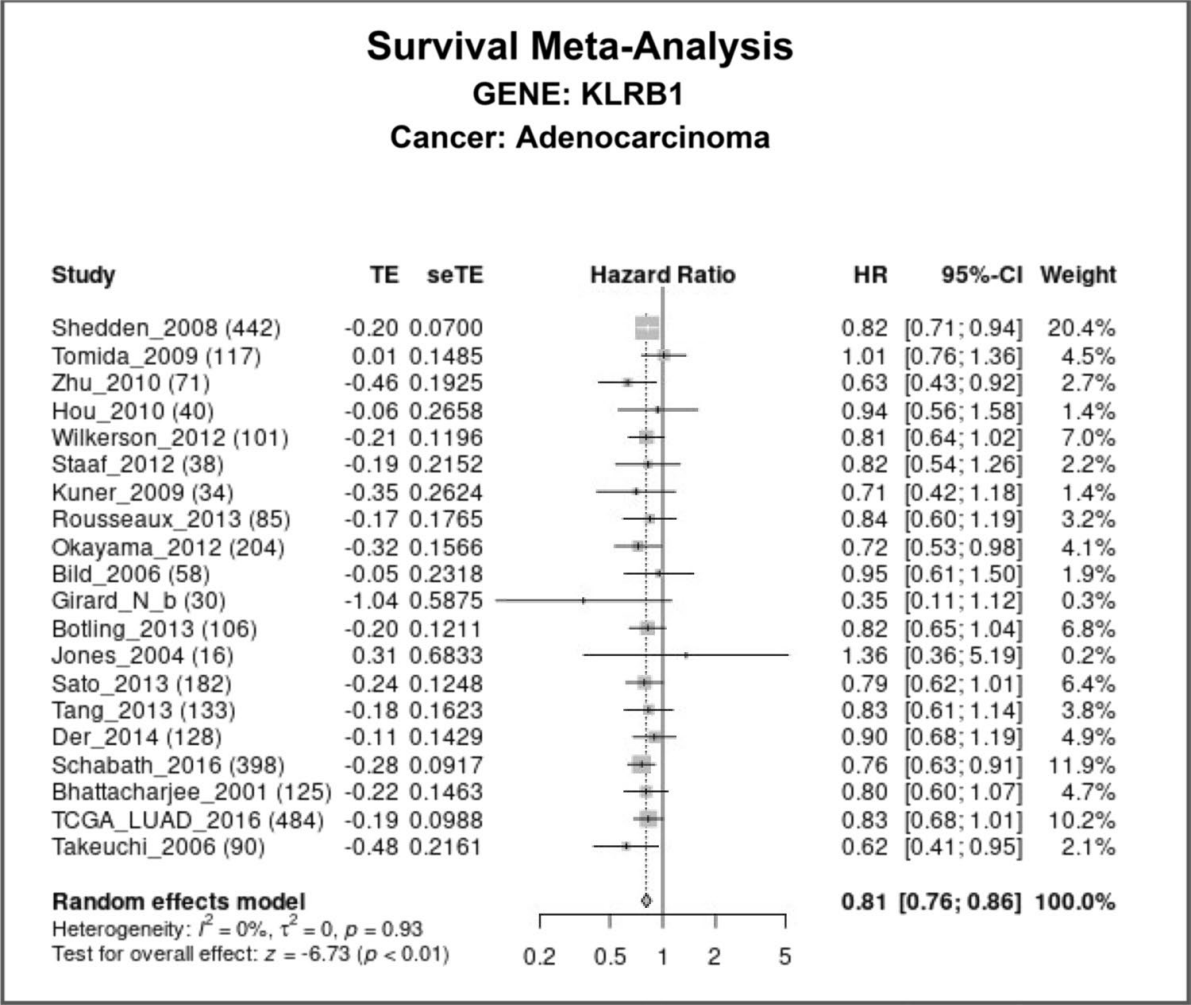
Meta-analysis of the LCE database depicting the prognostic effect of KLRB1 in LUAD. *LUAD* lung adenocarcinoma, *OS* overall survival, *LCE* lung cancer explorer.

ROC analysis showed that the area under the curve for KLRB1 was 0.734 for both the normal and LUAD tissues sourced from the TCGA database (Fig. 3A) and 0.694 for the normal and LUAD tissues sourced from the XENA database (Fig. 3B). In addition, K–M survival analysis using data from the TCGA database showed a significant correlation between reduced KLRB1 expression and the indicators of poor prognosis such as OS and DSS, but not PFI (Fig. 3C–E). Furthermore, analysis of data from the TCGA and GEO databases showed a significant correlation between reduced KLRB1 expression and poor OS in patients with LUAD (Fig. 4). Moreover, data analysis in the K-M plotter tool revealed that patients with LUAD with increased KLRB1 expression had a better survival time (Fig. S1). Therefore, preliminary findings suggest that KLRB1 may be a potential diagnostic and prognostic marker for patients with LUAD.

### Functions, mechanisms and networks associated with the genes co-expressed with KLRB1

**Figure 5:**
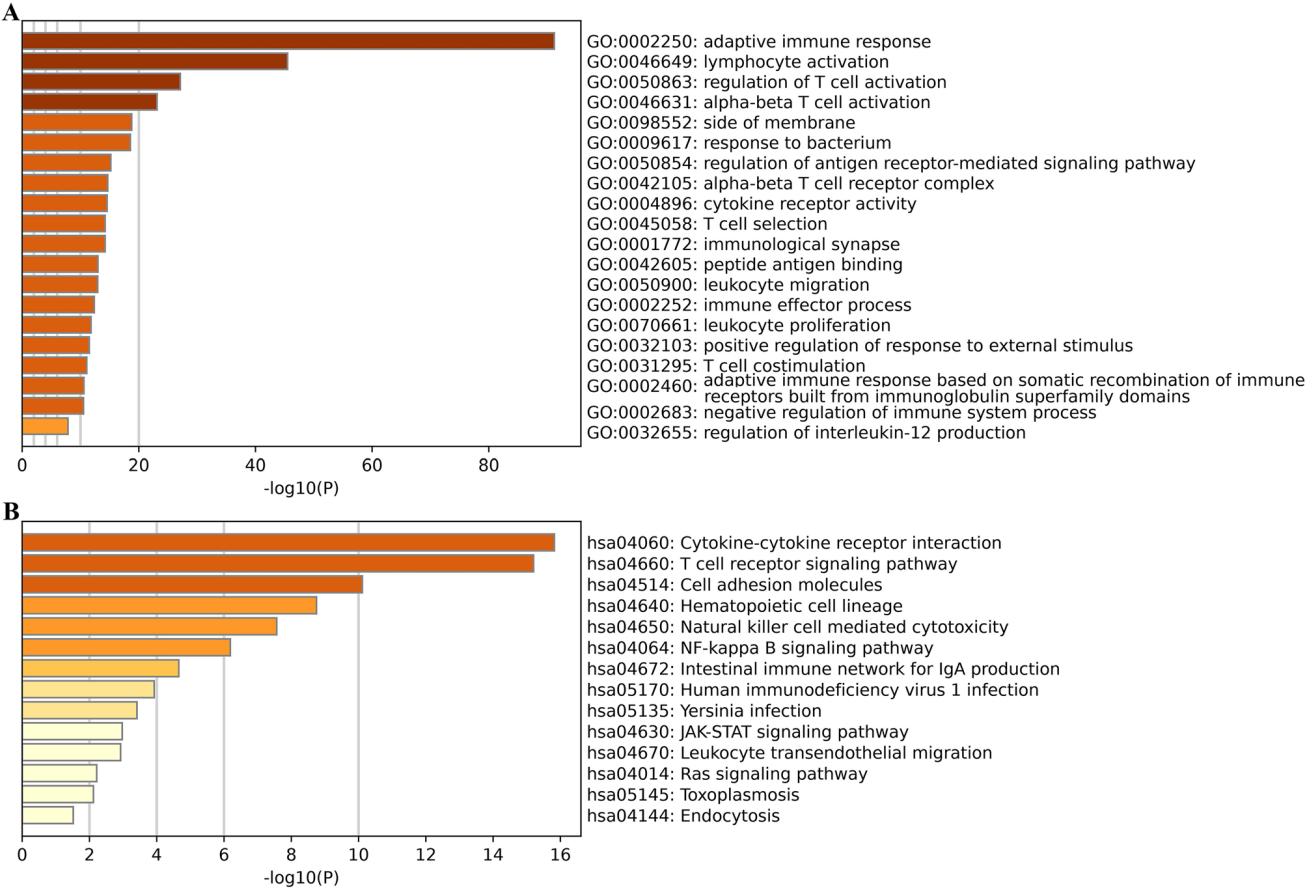
Functions and pathways associated with the genes co-expressed with KLRB1. (**A**) Functions; (**B**) Pathways.

**Figure 6:**
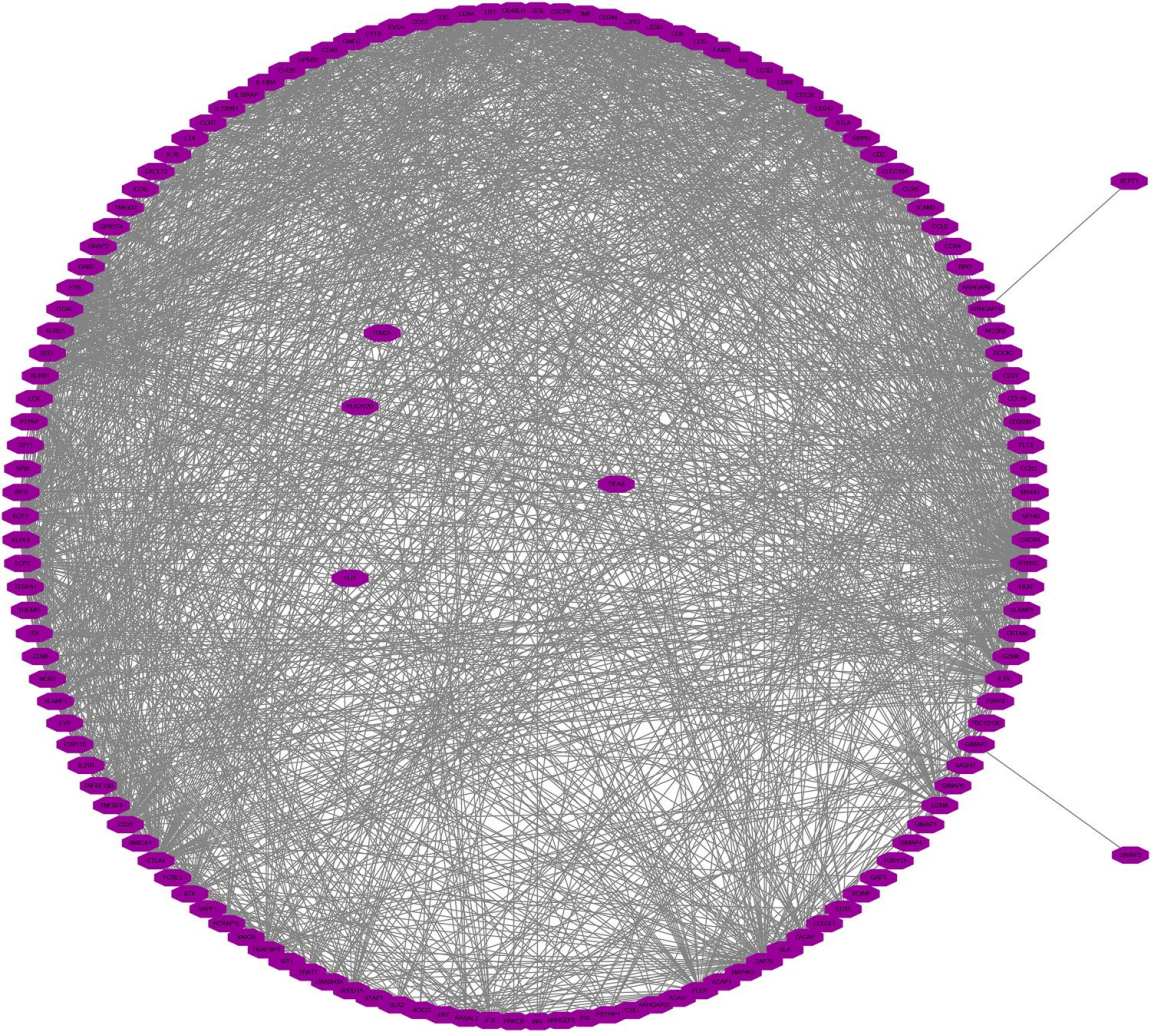
PPI network of the genes co-expressed with KLRB1. *PPI* protein–protein interaction.

A total of 210 genes were identified as co-expressed with KLRB1 when a correlation coefficient of 0.6 was used as the threshold. These 210 co-expressed genes were involved in T cell co-stimulation, leukocyte proliferation, immune effector processes, leukocyte migration, T cell selection, cytokine receptor activity, regulation of antigen receptor-mediated signaling pathway, regulation of T cell activation, and lymphocyte activation (Fig. 5A). Moreover, these genes were implicated in signaling pathways such as Ras, JAK-STAT, NF-kappa B, NK cell-mediated cytotoxicity, T cell receptor signaling, and others (Fig. 5B). GSEA revealed that KLRB1 was implicated in mechanisms such as T cell receptor, B cell receptor, NK cell-mediated cytotoxicity, Toll-like receptor, chemokine, NF-kappa B, PD-L1 expression and PD-1 checkpoint pathway in cancer, cell adhesion molecules, JAK-STAT, TNF, necroptosis, cell cycle, and other pathways (Table S1). Additionally, Fig. 6 shows the relationship network between KLRB1 co-expressed genes and genes.

### Overexpression of KLRB1 inhibits the growth and migration of LUAD cells

**Figure 7:**
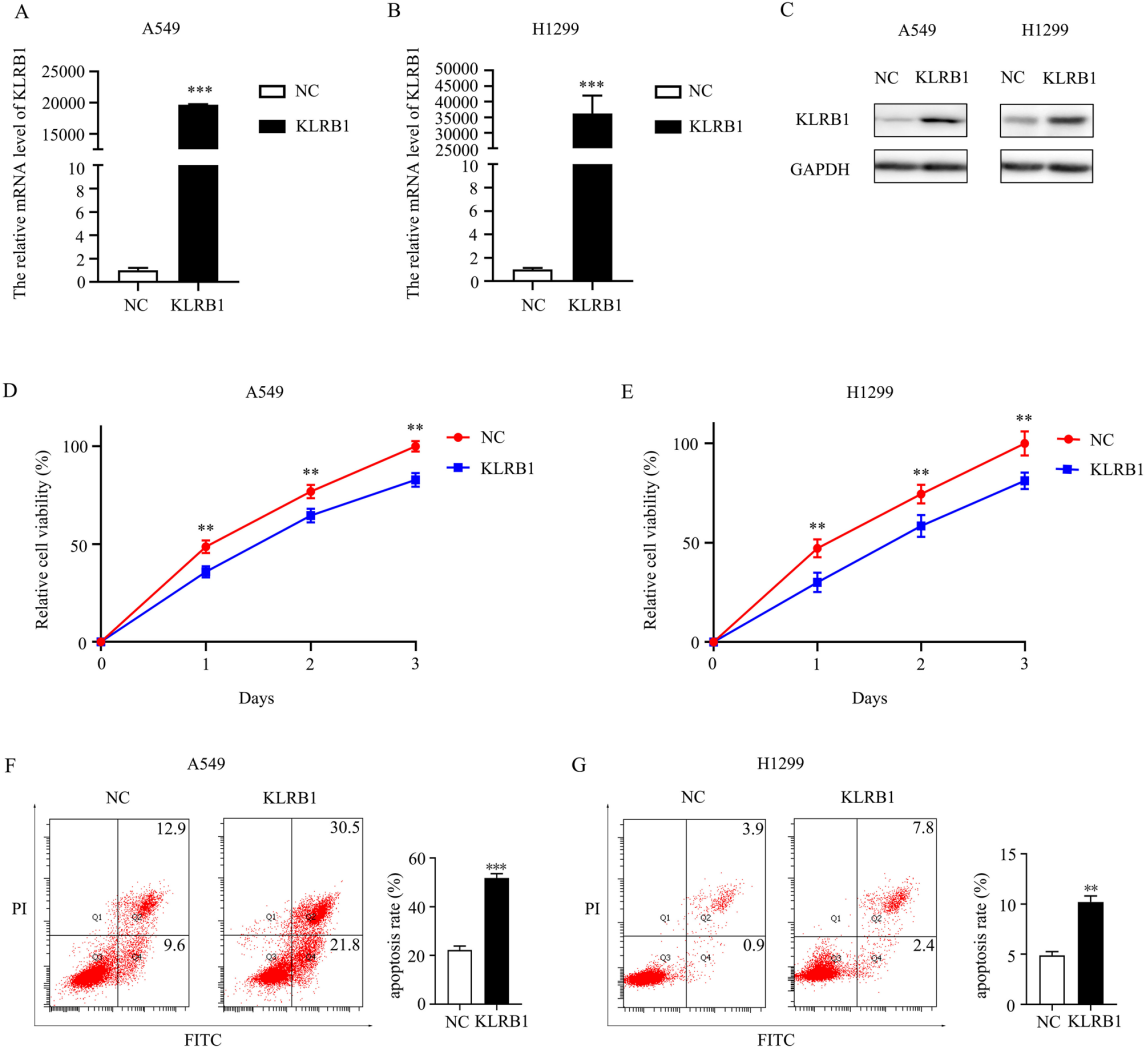
KLRB1 overexpression inhibits the growth and promotes the apoptosis of LUAD cells. (**A**–**C**) Results of PCR and Western blot analyses depicting the enhanced expression of KLRB1 in LUAD A549 and H1299 cells; (**D**–**F**) KLRB1 overexpression inhibits cancer cell proliferation and promotes apoptosis.

**Figure 8:**
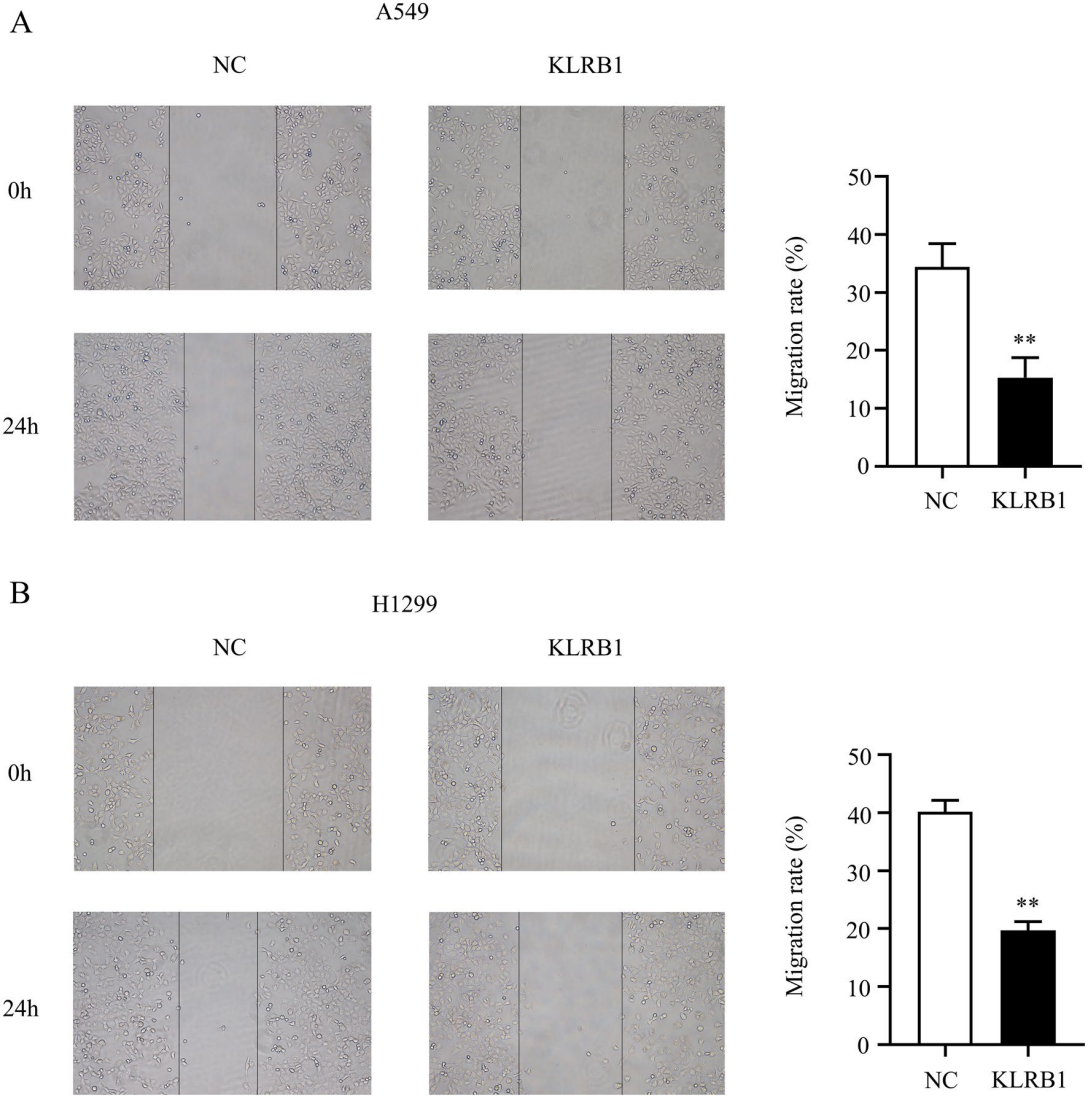
KLRB1 overexpression inhibits the cell migration capabilities of A549 and H1299 cells. (**A**) A549 cells; (**B**) H1299 cells.

**Figure 9:**
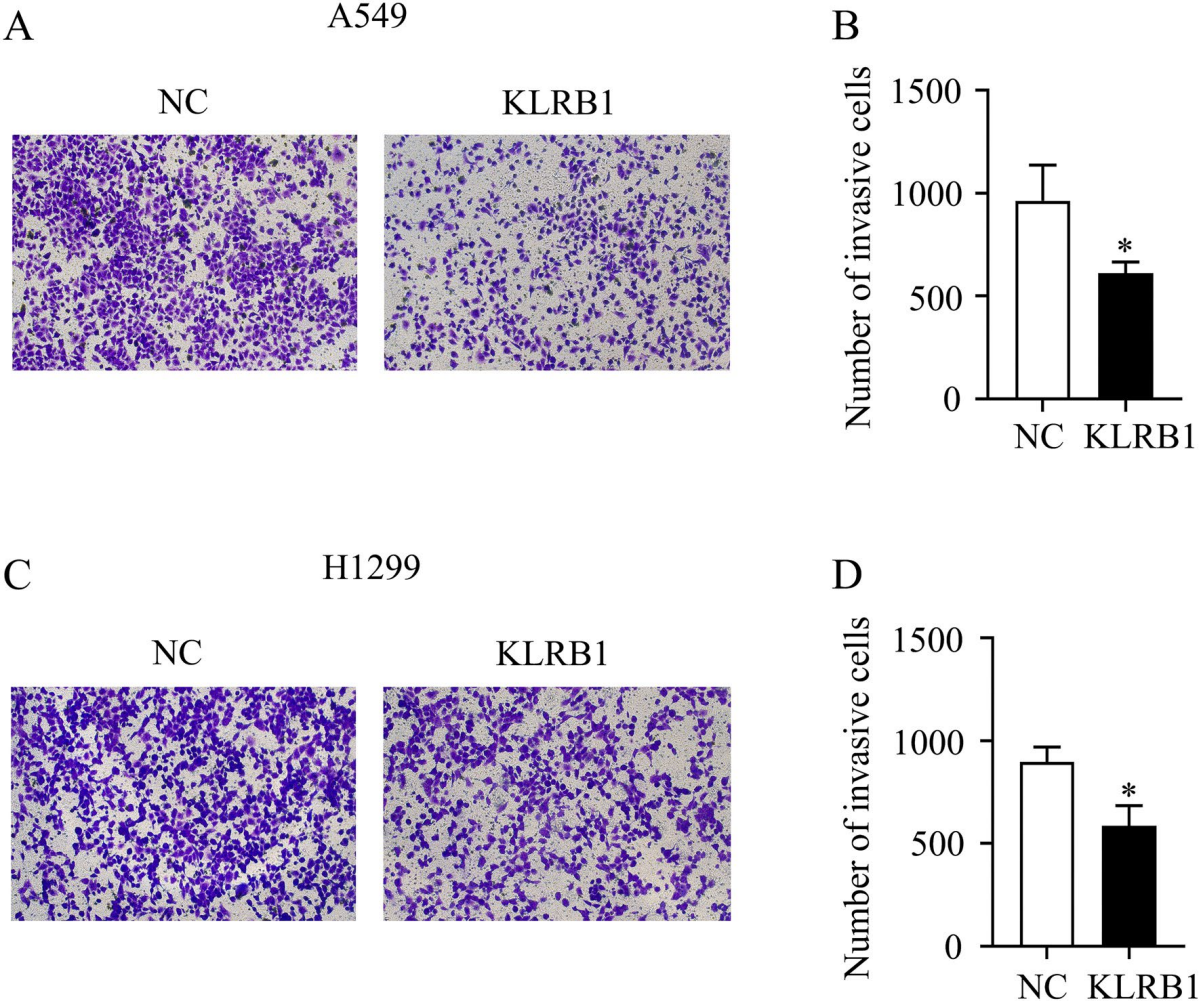
KLRB1 overexpression inhibits the cell invasion capabilities of A549 and H1299 cells. (**A**–**B**) A549 cells; (**C**–**D**) H1299 cells.

PCR and western blot analyses showed that adenovirus transfection of the KLRB1 gene led to the enhanced expression of KLRB1 in A549 and H1299 LUAD cells (Fig. 7A–C). In this study, we found that increased KLRB1 expression effectively inhibited the proliferation of A549 and H1299 cells, with statistically significant results observed at 24, 48, and 72 h post-transfection (Fig. 7D,E). Furthermore, an elevated KLRB1 expression level was found to promote apoptosis (Fig. 7F,G) while restraining the migration and invasion capabilities of the A549 and H1299 cells (Figs. 8 and 9).

### Down-regulation of KLRB1 expression is linked to changes in the tumor immune microenvironment of LUAD

**Figure 10:**
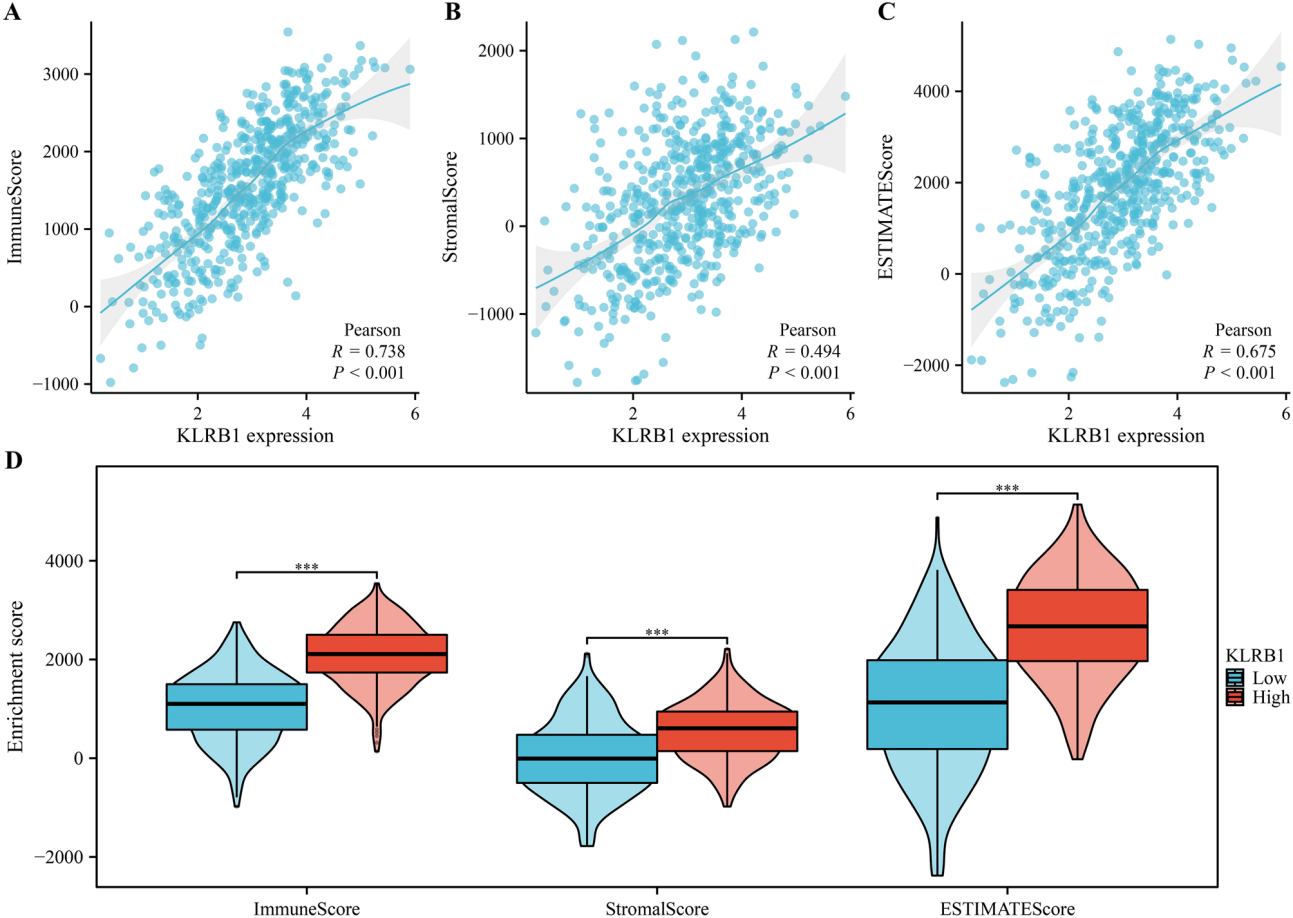
KLRB1 expression is linked to immune status in LUAD. (**A**) Immune score; (**B**) Stromal score; (**C**) Estimate score; (**D**) Immune, stromal, and estimate scores in KLRB1 high- and low- expression groups. *LUAD* lung adenocarcinoma.

**Figure 11:**
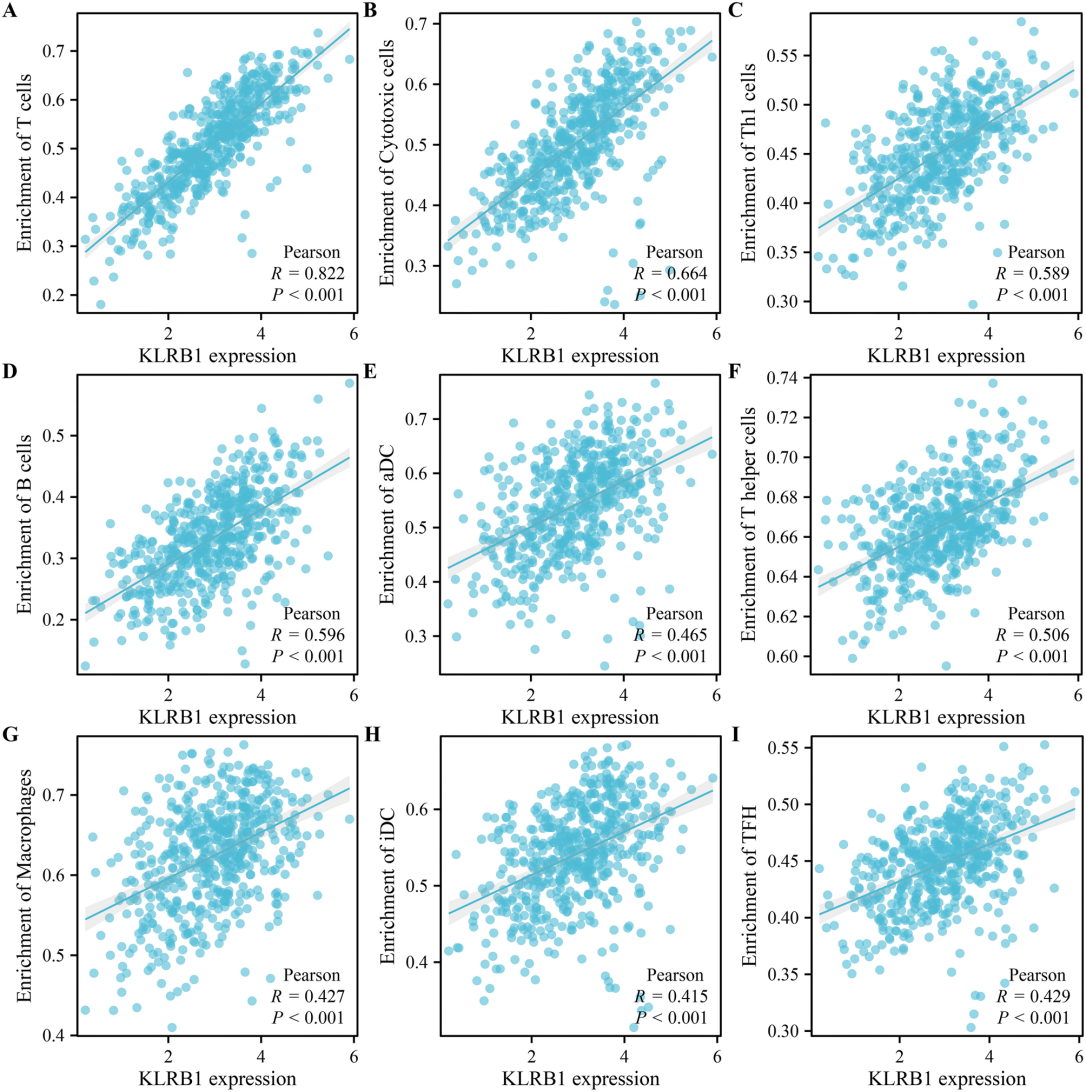
KLRB1 is associated with the levels of immune cells in LUAD. (**A**) T cells; (**B**) Cytotoxic T cells; (**C**) Th1 cells; (**D**) B cells; I aDC; (**F**) T helper cells; (**G**) Macrophages; (**H**) iDC; (**I**) TFH. *LUAD* lung adenocarcinoma.

**Figure 12:**
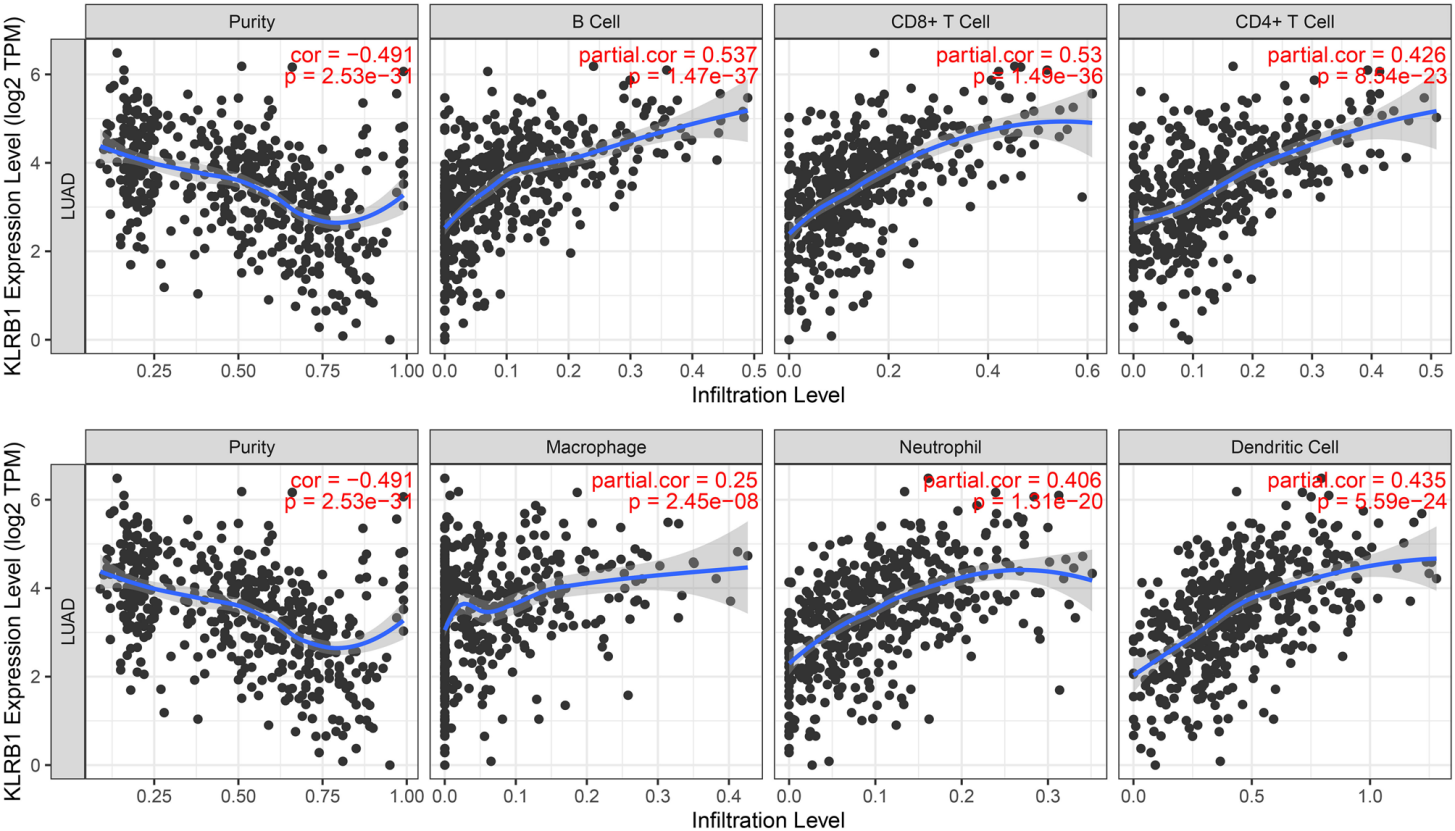
KLRB1 is associated with tumor purity and immune cell changes in LUAD using TIMER database. *LUAD* lung adenocarcinoma.

Analysis of the LUAD tissue data obtained from the TCGA database revealed that the expression level of KLRB1 was found to be significantly correlated with changes in the tumor immune microenvironment indicated via the stromal, immune, and estimate scores (Fig. 10A–C). Significant differences were noted in the stromal, immune, and estimate scores between the KLRB1-overexpression and -downregulation groups (Fig. 10D). The expression levels of KLRB1 were significantly correlated with the levels of immune cells such as T cells, cytotoxic T cells, B cells, Th1 cells, T helper cells, aDC, TFH, macrophages, iDC, DC, TReg, pDC, Tcm, mast cells, CD8^+^ T cells, CD56dim NK cells, Tem, neutrophils, eosinophils, and Th17 cells (Fig. 11 and Table 1). Additionally, the levels of aDC, B cells, CD8^+^ T cells, cytotoxic T cells, DC, eosinophils, iDC, macrophages, mast cells, neutrophils, CD56dim NK cells, pDC, T cells, T helper cells, Tcm, Tem, TFH, Th1 cells, Th17 cells, and TReg were found to be significantly different between the KLRB1-overexpression and -downregulation groups (Fig. S2). Furthermore, data analysis in the TIMER database revealed that the downregulation of KLRB1 expression was significantly positively correlated with tumor purity, and the levels of B cells, CD8^+^ T cells, CD4^+^ T cells, macrophages, neutrophils, and DC (Fig. 12). Preliminary evidence suggests that KLRB1 is associated with changes in the tumor immune microenvironment of LUAD.

**Table 1:** KLRB1 is associated with the levels of various immune cells in LUAD.

Gene	Immune cells	correlation coefficient	*P*
KLRB1	T cells	0.821517756	3.9937E−133
KLRB1	Cytotoxic cells	0.66362809	1.04353E−69
KLRB1	B cells	0.595931219	4.00549E−53
KLRB1	Th1 cells	0.588673533	1.42002E−51
KLRB1	T helper cells	0.505799247	2.36583E−36
KLRB1	aDC	0.465278254	2.61845E−30
KLRB1	TFH	0.428766293	1.63161E−25
KLRB1	Macrophages	0.426871538	2.79193E−25
KLRB1	iDC	0.414620188	8.3011E−24
KLRB1	DC	0.39428899	1.71265E−21
KLRB1	TReg	0.378771802	7.85837E−20
KLRB1	pDC	0.353058515	2.87854E−17
KLRB1	Tcm	0.338887	5.96295E−16
KLRB1	Mast cells	0.336456021	9.87578E−16
KLRB1	CD8 T cells	0.322902747	1.51728E−14
KLRB1	NK CD56dim cells	0.259168774	1.01108E−09
KLRB1	Tem	0.250288276	3.83124E−09
KLRB1	Neutrophils	0.23997177	1.69093E−08
KLRB1	Eosinophils	0.204662322	1.65709E−06
KLRB1	Th17 cells	0.123511949	0.004080819
KLRB1	Tgd	− 0.002677398	0.950550674
KLRB1	NK cells	− 0.009552366	0.824888846
KLRB1	NK CD56bright cells	− 0.026511387	0.539098184
KLRB1	Th2 cells	− 0.060389493	0.161498914

*LUAD* lung adenocarcinoma.

### Down-regulation of KLRB1 expression is linked to changes in the levels of the immune cell markers in LUAD

**Figure 13:**
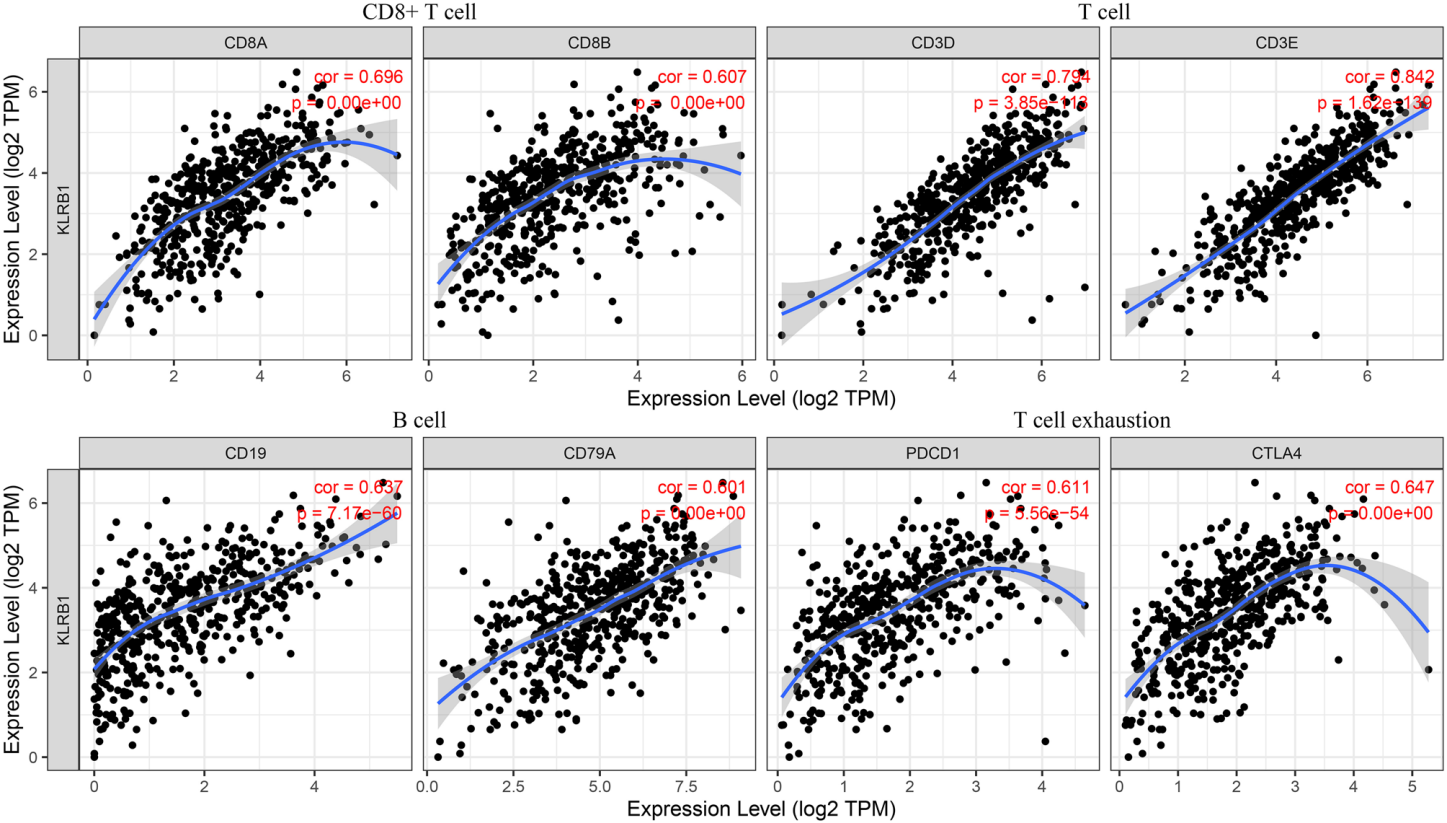
KLRB1 is associated with the expression of immune cell markers in LUAD under the condition of non-tumor purity. *LUAD* lung adenocarcinoma.

**Figure 14:**
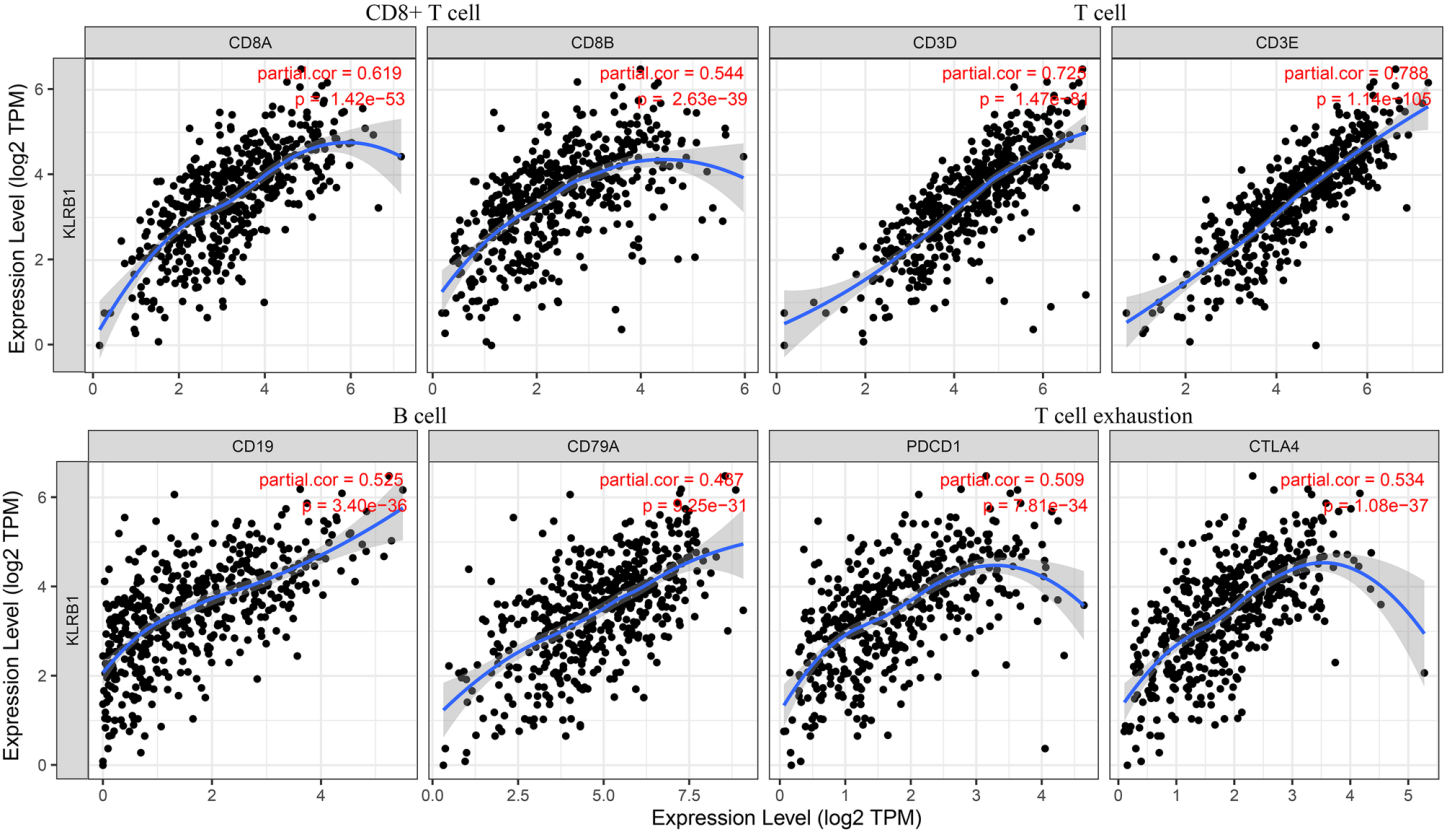
KLRB1 is associated with the expression of immune cell markers in LUAD under the condition of tumor purity. *LUAD* lung adenocarcinoma.

Data analysis in the TIMER database revealed that the down-regulation of KLRB1 expression was significantly correlated with changes in the expression levels of the immune cell markers in LUAD (Tables 2 and 3). Under non-tumor purity conditions, the downregulation of KLRB1 expression was significantly associated with changes in the expression levels of immune cell markers such as CD8A, CD8B, CD3D, CD3E, CD2, CD19, CD79A, CD86, PDCD1, CTLA4, LAG3, HAVCR2, GZMB, CSF1R, CCL2, CD68, IL10, NOS2, IRF5, PTGS2, CD163, VSIG4, MS4A4A, CEACAM8, ITGAM, CCR7, KIR2DL1, KIR2DL3, KIR2DL4, KIR3DL1, KIR3DL2, KIR3DL3, KIR2DS4, HLA-DPB1, HLA-DQB1, HLA-DRA, HLA-DPA1, CD1C, ITGAX, TBX21, STAT4, STAT1, IFNG, TNF, GATA3, STAT6, STAT5A, IL13, IL21, IL17A, FOXP3, CCR8, STAT5B, and TGFB1 (Table 3 and Fig. 13). In the tumor purity condition, the down-regulation of KLRB1 expression was significantly correlated with changes in the expression levels of immune cell markers such as CD3E, CD2, CD3D, CCR7, CD8A, TBX21, CD8B, CTLA4, CD19, HLA-DPB1, PDCD1, HLA-DRA, HLA-DPA1, CD79A, CCR8, STAT4, FOXP3, IFNG, CD86, HAVCR2, STAT5A, MS4A4A, LAG3, IL10, GZMB, GATA3, CD1C, IL17A, CSF1R, ITGAX, IL21, HLA-DQB1, VSIG4, CD68, STAT1, CD163, STAT5B, ITGAM, KIR3DL2, KIR2DL3, KIR2DS4, KIR2DL4, KIR3DL1, CEACAM8, KIR2DL1, TNF, TGFB1, PTGS2, IRF5, STAT6, CCL2, IL13, and KIR3DL3 (Table 4 and Fig. 14). Furthermore, data analysis in the GEPIA database revealed that the down-regulation of KLRB1 expression had a strong correlation with the expression levels of most of the immune cell markers (Table 4).

**Table 2:** KLRB1 is associated with the expression of immune cell markers in LUAD under non-tumor purity condition.

Gene	Markers	Correlation coefficient	*P*
KLRB1	CD8A	0.696089094	0
KLRB1	CD8B	0.606850322	0
KLRB1	CD3D	0.794361069	3.85E−113
KLRB1	CD3E	0.841867204	1.62E−139
KLRB1	CD2	0.837471495	9.99E−137
KLRB1	CD19	0.636604963	7.17E−60
KLRB1	CD79A	0.601164416	0
KLRB1	CD86	0.540334267	0
KLRB1	CSF1R	0.450426946	4.27E−27
KLRB1	CCL2	0.273691526	3.16E−10
KLRB1	CD68	0.404186152	0
KLRB1	IL10	0.483040205	1.83E−31
KLRB1	NOS2	0.121462682	0.005780943
KLRB1	IRF5	0.281950674	8.78E−11
KLRB1	PTGS2	− 0.134564548	0.002228322
KLRB1	CD163	0.405472246	0
KLRB1	VSIG4	0.386272033	0
KLRB1	MS4A4A	0.491912255	0
KLRB1	CEACAM8	0.192279595	1.11E−05
KLRB1	ITGAM	0.392983107	0
KLRB1	CCR7	0.72803129	0
KLRB1	KIR2DL1	0.229398637	1.41E−07
KLRB1	KIR2DL3	0.320766679	8.67E−14
KLRB1	KIR2DL4	0.284657317	4.68E−11
KLRB1	KIR3DL1	0.256325198	3.60E−09
KLRB1	KIR3DL2	0.337197736	3.69E−15
KLRB1	KIR3DL3	0.11568175	0.008596815
KLRB1	KIR2DS4	0.275350705	2.06E−10
KLRB1	HLA-DPB1	0.612901583	1.93E−54
KLRB1	HLA-DQB1	0.40539345	8.60E−22
KLRB1	HLA-DRA	0.60141357	0
KLRB1	HLA-DPA1	0.587421074	0
KLRB1	CD1C	0.417384852	3.97E−23
KLRB1	NRP1	0.063755158	0.148474503
KLRB1	ITGAX	0.443675581	3.00E−26
KLRB1	TBX21	0.670463772	0
KLRB1	STAT4	0.5703372	9.08E−46
KLRB1	STAT1	0.372713008	0
KLRB1	IFNG	0.505420938	9.58E−35
KLRB1	TNF	0.336113676	6.08E−15
KLRB1	GATA3	0.464874823	5.66E−29
KLRB1	STAT6	0.115265675	0.008871062
KLRB1	STAT5A	0.525306161	7.17E−38
KLRB1	IL13	0.201732827	3.94E−06
KLRB1	BCL6	0.026870388	0.542789285
KLRB1	IL21	0.344745005	8.10E−16
KLRB1	STAT3	0.037214882	0.399351558
KLRB1	IL17A	0.376550006	8.56E−19
KLRB1	FOXP3	0.564082941	0
KLRB1	CCR8	0.568747421	1.81E−45
KLRB1	STAT5B	0.268663124	5.78E−10
KLRB1	TGFB1	0.279492872	1.29E−10
KLRB1	PDCD1	0.610800789	5.56E−54
KLRB1	CTLA4	0.647093309	0
KLRB1	LAG3	0.465435189	0
KLRB1	HAVCR2	0.539631435	0
KLRB1	GZMB	0.461949868	1.38E−28

*LUAD* lung adenocarcinoma.

**Table 3:** KLRB1 is linked to the expression of immune cell markers in LUAD under tumor purity condition.

Gene	Markers	Correlation coefficient	*P*
KLRB1	CD3E	0.788392436	1.14E−105
KLRB1	CD2	0.785961308	1.35E−104
KLRB1	CD3D	0.725050421	1.47E−81
KLRB1	CCR7	0.634758447	5.87E−57
KLRB1	CD8A	0.619450533	1.42E−53
KLRB1	TBX21	0.581687517	5.65E−46
KLRB1	CD8B	0.543918632	2.63E−39
KLRB1	CTLA4	0.534000677	1.08E−37
KLRB1	CD19	0.524502171	3.40E−36
KLRB1	HLA-DPB1	0.524148861	3.86E−36
KLRB1	PDCD1	0.508883577	7.81E−34
KLRB1	HLA-DRA	0.506724777	1.62E−33
KLRB1	HLA-DPA1	0.503483153	4.80E−33
KLRB1	CD79A	0.487260588	9.25E−31
KLRB1	CCR8	0.461868067	2.03E−27
KLRB1	STAT4	0.46148047	2.27E−27
KLRB1	FOXP3	0.437821441	1.67E−24
KLRB1	IFNG	0.415737069	5.03E−22
KLRB1	CD86	0.411669233	1.38E−21
KLRB1	HAVCR2	0.409010552	2.64E−21
KLRB1	STAT5A	0.407363699	3.94E−21
KLRB1	MS4A4A	0.367700355	3.14E−17
KLRB1	LAG3	0.36004361	1.55E−16
KLRB1	IL10	0.356280318	3.35E−16
KLRB1	GZMB	0.343881216	3.94E−15
KLRB1	GATA3	0.339381517	9.37E−15
KLRB1	CD1C	0.336280592	1.69E−14
KLRB1	IL17A	0.321292942	2.66E−13
KLRB1	CSF1R	0.319799453	3.48E−13
KLRB1	ITGAX	0.293029061	3.22E−11
KLRB1	IL21	0.29024166	5.03E−11
KLRB1	HLA-DQB1	0.281145777	2.08E−10
KLRB1	VSIG4	0.278635636	3.05E−10
KLRB1	CD68	0.277027589	3.89E−10
KLRB1	STAT1	0.275636525	4.80E−10
KLRB1	CD163	0.273274092	6.82E−10
KLRB1	STAT5B	0.264873872	2.33E−09
KLRB1	ITGAM	0.26181879	3.60E−09
KLRB1	KIR3DL2	0.256804547	7.26E−09
KLRB1	KIR2DL3	0.237873003	9.03E−08
KLRB1	KIR2DS4	0.212183507	2.00E−06
KLRB1	KIR2DL4	0.202182104	6.05E−06
KLRB1	KIR3DL1	0.198551363	8.93E−06
KLRB1	CEACAM8	0.191340242	1.89E−05
KLRB1	KIR2DL1	0.175930779	8.60E−05
KLRB1	TNF	0.173559718	0.00010737
KLRB1	TGFB1	0.160656538	0.000341773
KLRB1	PTGS2	− 0.152169222	0.000699208
KLRB1	IRF5	0.149590019	0.000862951
KLRB1	STAT6	0.147279031	0.001039079
KLRB1	CCL2	0.137961199	0.002139491
KLRB1	IL13	0.116705783	0.009498267
KLRB1	KIR3DL3	0.099071694	0.027836821
KLRB1	STAT3	0.059989277	0.183585893
KLRB1	NOS2	0.019902975	0.659329089
KLRB1	NRP1	0.017602008	0.696635556
KLRB1	BCL6	0.016900764	0.708157306

*LUAD* lung adenocarcinoma.

**Table 4:** Data analysis in the TIMER database depicting the link between KLRB1 and the expression of immune cell markers in LUAD.

Gene	Markers	Correlation coefficient	*P*
KLRB1	CD3E	0.77	0
KLRB1	CD2	0.76	0
KLRB1	CD3D	0.7	0
KLRB1	CCR7	0.67	0
KLRB1	CD8A	0.57	0
KLRB1	TBX21	0.12	0.0064
KLRB1	CD8B	0.41	0
KLRB1	CTLA4	0.44	0
KLRB1	CD19	0.57	0
KLRB1	HLA-DPB1	0.45	0
KLRB1	PDCD1	0.44	0
KLRB1	HLA-DRA	0.49	0
KLRB1	HLA-DPA1	0.46	0
KLRB1	CD79A	0.51	0
KLRB1	CCR8	0.46	0
KLRB1	STAT4	0.34	2.2e−14
KLRB1	FOXP3	0.51	0
KLRB1	IFNG	0.4	0
KLRB1	CD86	0.46	0
KLRB1	HAVCR2	0.43	0
KLRB1	STAT5A	0.46	0
KLRB1	MS4A4A	0.33	2e−13
KLRB1	LAG3	0.24	1.1e−07
KLRB1	IL10	0.33	5.6e−14
KLRB1	GZMB	0.29	5.6e−11
KLRB1	GATA3	0.001	0.98
KLRB1	CD1C	0.29	1.5e−10
KLRB1	IL17A	0.28	5.7e−10
KLRB1	CSF1R	0.32	9e−13
KLRB1	ITGAX	0.33	5e−14
KLRB1	IL21	0.43	0
KLRB1	HLA-DQB1	0.25	4.2e−08
KLRB1	VSIG4	0.24	5e−08
KLRB1	CD68	0.26	7.6e−09
KLRB1	STAT1	0.27	3e−09
KLRB1	CD163	0.22	9.3e−07
KLRB1	STAT5B	0.21	2.5e−06
KLRB1	ITGAM	0.23	2.3e−07
KLRB1	KIR3DL2	− 0.017	0.72
KLRB1	KIR2DL3	0.055	0.23
KLRB1	KIR2DS4	0.037	0.42
KLRB1	KIR2DL4	0.13	0.0053
KLRB1	KIR3DL1	0.085	0.062
KLRB1	CEACAM8	0.016	0.73
KLRB1	KIR2DL1	− 0.039	0.39
KLRB1	TNF	0.22	7.3e−07
KLRB1	TGFB1	0.15	0.0012
KLRB1	PTGS2	− 0.14	0.0018
KLRB1	IRF5	0.18	8.5e−05
KLRB1	STAT6	0.067	0.14
KLRB1	CCL2	0.13	0.0047
KLRB1	IL13	0.25	4.3e−08
KLRB1	KIR3DL3	0.12	0.011

*LUAD* lung adenocarcinoma.

## Discussion

Upon immune activation, NK cells, a type of lymphocyte, play a vital role in cytotoxicity and secrete cytokines that participate in the process of disease. KLRB1, an NK cell receptor, plays an essential role in regulating the ability of the immune system to fight exogenous and self-diseases and is significantly associated with cancer progression^8–11^. However, the specific roles and mechanisms of KLRB1 in lung cancer remain unclear. Recent research has shown that certain tumor suppressor genes can significantly inhibit the occurrence and development of lung cancer and that they are strongly correlated with poor prognosis^26–28^. For example, progestin and adipoQ receptor family member 3 (PAQR3) is overexpressed in LUAD and is strongly correlated with prognosis in LUAD patients. The overexpression of PAQR3 inhibits the growth, invasion, and migration of LUAD cells and promotes apoptosis^26^. Notably, our study revealed the weak expression of KLRB1 in LUAD tissues. However, the inhibition of KLRB1 expression was linked to several clinical factors, including T stage, clinical stage, distant metastasis, gender, diagnosis, and poor prognosis. These findings suggest that KLRB1 may function as a tumor suppressor gene in LUAD and that it can serve as a potential target molecule for the treatment of LUAD, thereby offering new hope to patients with LUAD.

The occurrence and development of LUAD involve a variety of defects in the mechanisms underlying cancer development and progression such as the JAK-STAT, Nf-kB, PD-L1, and PD-1 pathways^26,29–31^. For example, proteasome 20S subunit alpha 5 (PSMA5) is associated with intracellular protein degradation and is significantly up-regulated in LUAD cells and tissues. In addition, the upregulation of PSMA5 expression is positively correlated with lymph node metastasis and poor prognosis in patients with LUAD. Conversely, the inhibition of PSMA5 expression inhibits the growth and metastasis of LUAD cells in vitro and in vivo, induces apoptosis, and promotes the sensitivity of LUAD cells to cisplatin via the inactivation of the JAK/STAT signaling pathway^29^. In addition, the inhibition of cyclin-dependent kinase 5 (CDK5) expression in the Lewis lung cancer cells of mice did not affect cell proliferation. In a mouse tumor model, the inhibition of CDK5 expression or the combination of CDK5 inhibition with anti-PD-L1 therapy can significantly inhibit tumor growth and lead to increased levels of CD3^+^, CD4^+^, and CD8^+^ T cells in the spleen and decreased PD-1 expression in the CD4^+^ T cells^30^. Moreover, KEGG analysis and GSEA revealed that KLRB1 is implicated in the JAK-STAT, NF-kB, PD-L1, and PD-1 pathways, which further confirmed the significant roles of KLRB1 in the progress of LUAD. However, this needs further verification using Western blot in the future.

NK cells play a critical role in the tumor immune microenvironment and improving the cytotoxic functions of NK cells is crucial for the effective treatment of cancer, particularly LUAD^32,33^. STK11, which is expressed in both the tissues and cells of LUAD, can impact NK cell infiltration and activity. Overexpression of STK11 results in the reduced infiltration of NK cells, significantly decreased proliferation of LUAD cells, and the induction of apoptosis. Conversely, the reduced expression of STK11 results in reduced chemotaxis and NK cell activity while promoting the growth and proliferation of LUAD cells in vivo^32^. KLRB1, a receptor on NK cells, plays a critical role in regulating NK cell function. Moreover, KLRB1 has a significant relationship with LUAD purity and the tumor immune microenvironment of LUAD. KLRB1 expression is correlated with the levels of various immune cells, including T cells, cytotoxic T cells, B cells, Th1 cells, T helper cells, aDC, TFH, macrophages, iDC, DC, Treg, pDC, Tcm, mast cells, CD56dim NK cells, Tem, neutrophils, eosinophils, Th17 cells, CD8^+^ T cells, and CD4^+^ T cells. It is also associated with the expression levels of a multitude of immune cell markers such as CD2, CD3D, CCR7, TBX21, CTLA4, PDCD1, and GZMB, as well as various cytokines and chemokines. According to GO analysis and GSEA, the KLRB1 gene is involved in regulating various aspects of immune cell function, including T cell activation, differentiation, and costimulation, as well as signaling pathways for B cells and chemokines. Additionally, KLRB1 negatively regulates excessive immune responses mediated by T cells and B cells and down-regulates the chemotaxis of NK cells. These findings support the important role of KLRB1 in regulating NK cells and shaping the tumor immune microenvironment of LUAD.

This study reveals the roles and signaling mechanisms of the NK cell marker, KLRB1, in LUAD, thus providing a novel candidate target for the treatment of patients with LUAD. However, this study has some shortcomings. In the future, clinical tissue samples should be collected and studied to verify the expression of KLRB1 in LUAD and explore its potential clinical values in LUAD. In addition, cell models should be constructed to explore the roles and mechanisms of KLRB1 in the progression of LUAD in vivo. In short, reduced KLRB1 expression has a significant positive correlation with the diagnosis, poor prognosis, and cancer immunity of patients with LUAD. These results suggest that KLRB1 may serve as a potential therapeutic target for patients with LUAD.

### Supplementary Information

Supplementary Information.

## Data Availability

The datasets used in this study can be accessed through the TCGA (https://www.cancer.gov/ccg/) and XENA (https://xena.ucsc.edu/) databases, and the experimental data can be obtained from the corresponding author.
