# Supplementary material for: Down-regulation of KLRB1 is associated with increased cell growth, metastasis, poor prognosis, as well as a dysfunctional immune microenvironment in LUAD
Source: Sci Rep. 2024 May 23;14:11782. doi: 10.1038/s41598-024-60414-x (PMC11116539; doi:10.1038/s41598-024-60414-x)
Supplement: Supplementary file 1 — Supplementary Information. [file 41598_2024_60414_MOESM1_ESM.docx]

**Supplementary figure**


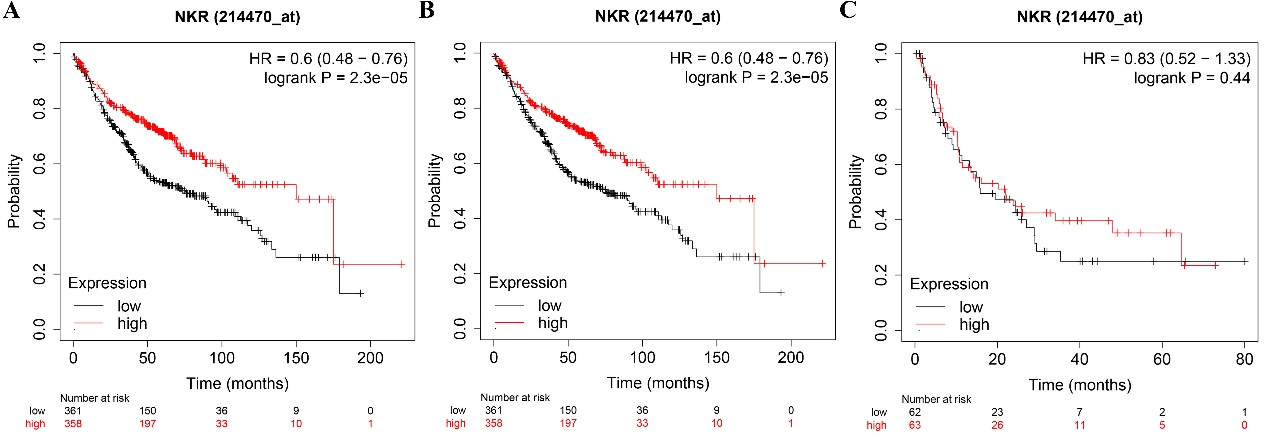


Figure S1. Survival analysis in the Kaplan-Meier plotter database depicting the prognostic effect of KLRB1 in LUAD. (A) OS; (B) FP; (C) PPS.

LUAD, lung adenocarcinoma; OS, overall survival.


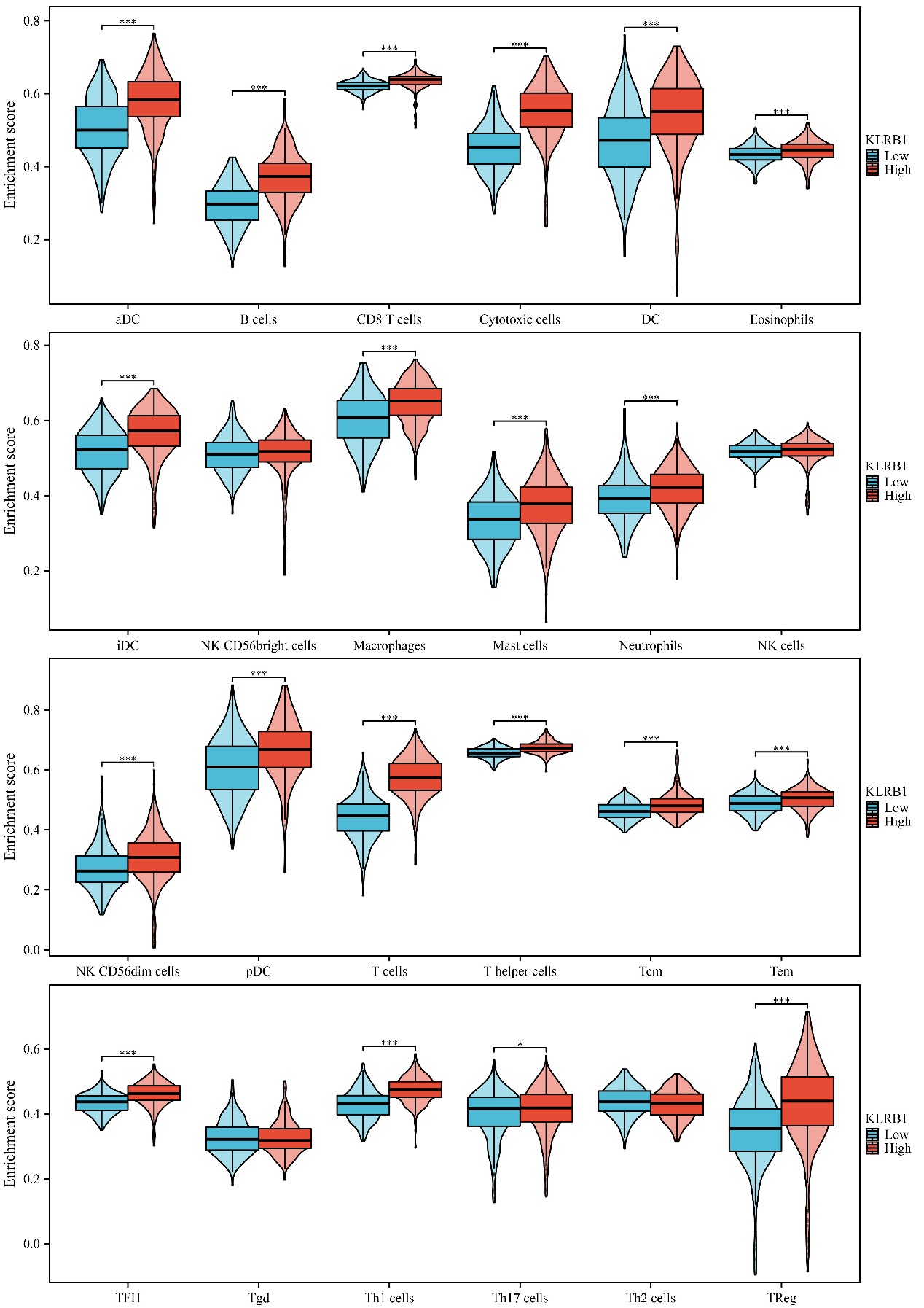


Figure S2. Immune cell levels in the KLRB1 high- and low- expression groups of LUAD.

LUAD, lung adenocarcinoma.

**Supplementary table**

Table S1. GSEA depicting the pathways associated with KLRB1.

| GSEA | Description | Size | Adjust P |
| --- | --- | --- | --- |
| hsa05310 | Asthma | 22 | 1.43E-07 |
| hsa05330 | Allograft rejection | 32 | 3.01E-09 |
| hsa05340 | Primary immunodeficiency | 36 | 3.01E-09 |
| hsa04672 | Intestinal immune network for IgA production | 41 | 3.01E-09 |
| hsa05320 | Autoimmune thyroid disease | 33 | 2.21E-07 |
| hsa05321 | Inflammatory bowel disease | 56 | 3.01E-09 |
| hsa05322 | Systemic lupus erythematosus | 52 | 4.03E-09 |
| hsa04061 | Viral protein interaction with cytokine and cytokine receptor | 95 | 3.01E-09 |
| hsa04640 | Hematopoietic cell lineage | 89 | 3.01E-09 |
| hsa05144 | Malaria | 47 | 1.89E-07 |
| hsa04659 | Th17 cell differentiation | 100 | 3.01E-09 |
| hsa05332 | Graft-versus-host disease | 37 | 4.10E-05 |
| hsa05416 | Viral myocarditis | 57 | 6.71E-08 |
| hsa04658 | Th1 and Th2 cell differentiation | 85 | 3.01E-09 |
| hsa05143 | African trypanosomiasis | 34 | 0.000366576 |
| hsa05140 | Leishmaniasis | 73 | 1.48E-07 |
| hsa04612 | Antigen processing and presentation | 70 | 4.23E-07 |
| hsa04660 | T cell receptor signaling pathway | 99 | 9.17E-09 |
| hsa04662 | B cell receptor signaling pathway | 78 | 4.86E-07 |
| hsa04650 | Natural killer cell mediated cytotoxicity | 108 | 4.03E-09 |
| hsa05323 | Rheumatoid arthritis | 87 | 5.71E-07 |
| hsa04380 | Osteoclast differentiation | 124 | 4.03E-09 |
| hsa04620 | Toll-like receptor signaling pathway | 86 | 5.17E-06 |
| hsa05152 | Tuberculosis | 159 | 3.01E-09 |
| hsa04062 | Chemokine signaling pathway | 186 | 3.01E-09 |
| hsa04975 | Fat digestion and absorption | 40 | 0.007465211 |
| hsa04613 | Neutrophil extracellular trap formation | 107 | 3.93E-07 |
| hsa05133 | Pertussis | 75 | 8.15E-05 |
| hsa04064 | NF-kappa B signaling pathway | 100 | 8.19E-06 |
| hsa05145 | Toxoplasmosis | 110 | 4.65E-06 |
| hsa04940 | Type I diabetes mellitus | 40 | 0.019098143 |
| hsa05142 | Chagas disease | 98 | 2.12E-05 |
| hsa05235 | PD-L1 expression and PD-1 checkpoint pathway in cancer | 86 | 6.87E-05 |
| hsa04610 | Complement and coagulation cascades | 81 | 0.000178818 |
| hsa04514 | Cell adhesion molecules | 142 | 4.68E-08 |
| hsa04060 | Cytokine-cytokine receptor interaction | 251 | 3.01E-09 |
| hsa04145 | Phagosome | 144 | 3.05E-07 |
| hsa04625 | C-type lectin receptor signaling pathway | 102 | 4.44E-05 |
| hsa05169 | Epstein-Barr virus infection | 184 | 1.17E-08 |
| hsa04623 | Cytosolic DNA-sensing pathway | 46 | 0.01787532 |
| hsa04979 | Cholesterol metabolism | 49 | 0.021574315 |
| hsa05164 | Influenza A | 149 | 5.17E-06 |
| hsa05171 | Coronavirus disease - COVID-19 | 207 | 2.81E-08 |
| hsa04664 | Fc epsilon RI signaling pathway | 63 | 0.033124747 |
| hsa05166 | Human T-cell leukemia virus 1 infection | 213 | 4.86E-07 |
| hsa05162 | Measles | 122 | 0.000504058 |
| hsa04630 | JAK-STAT signaling pathway | 133 | 0.000366576 |
| hsa04668 | TNF signaling pathway | 111 | 0.0017548 |
| hsa04611 | Platelet activation | 121 | 0.000937675 |
| hsa05135 | Yersinia infection | 134 | 0.001083497 |
| hsa04217 | Necroptosis | 117 | 0.006911396 |
| hsa05150 | Staphylococcus aureus infection | 82 | 0.038480708 |
| hsa05417 | Lipid and atherosclerosis | 197 | 8.15E-05 |
| hsa05170 | Human immunodeficiency virus 1 infection | 193 | 0.000366576 |
| hsa05146 | Amoebiasis | 99 | 0.036422166 |
| hsa05167 | Kaposi sarcoma-associated herpesvirus infection | 178 | 0.001330238 |
| hsa04670 | Leukocyte transendothelial migration | 107 | 0.022073726 |
| hsa05163 | Human cytomegalovirus infection | 205 | 0.004728597 |
| hsa04261 | Adrenergic signaling in cardiomyocytes | 146 | 0.042862381 |
| hsa03040 | Spliceosome | 134 | 0.034666343 |
| hsa04110 | Cell cycle | 125 | 0.016704126 |
| hsa04080 | Neuroactive ligand-receptor interaction | 299 | 1.75E-05 |
| hsa05033 | Nicotine addiction | 38 | 0.040473456 |
| hsa04742 | Taste transduction | 63 | 0.002349118 |
| hsa04950 | Maturity onset diabetes of the young | 26 | 0.008376828 |

Note: GSEA, Gene set enrichment analysis.
